# Norwegian translation and adaptation of the INT4 version of the Adult Social Care Outcomes Toolkit (ASCOT)

**DOI:** 10.1007/s11136-026-04209-9

**Published:** 2026-04-01

**Authors:** Ingeborg Strømseng Sjetne, Nick Smith, Ann-Marie Towers, Åste Renolen, Siv Fladsrud Magnussen, Lisa Victoria Burrell, Marijke Veenstra, Maren K. Raknes Sogstad

**Affiliations:** 1 Division of Health Services, Norwegian Institute of Public Health, Oslo, Norway; 2 Centre for Health Service Studies, The University of Kent, Canterbury, Kent, CT2 7NF UK; 3 Health and Social Care Workforce Research Unit, The Policy Institute, King’s College London, 22 Kingsway, London, WC2B 6LE UK; 4 Centre for Development of Institutional and Home Care Services, Innlandet County, Norway; 5 Centre for Care Research, Norwegian University of Science and Technology (NTNU), Gjøvik, Norway; 6 Health Services Research Unit, Akershus University Hospital, Lørenskog, Norway

**Keywords:** ASCOT, Long-term care, Quality of life, Measurement, Norway, User perspective

## Abstract

**Purpose:**

In Norway, no standardized instrument has been available to measure outcomes of long-term care (LTC) from the perspective of the service users. The Adult Social Care Outcomes Toolkit (ASCOT), developed in England, was designed for this purpose. ASCOT is a suite of tools designed that assess outcomes from the perspective of different groups receiving LTC. This article describes the translation and cultural adaptation of the four-level interview version (INT4) for use in Norwegian LTC services.

**Methods:**

The translation followed international guidelines and included two forward translations and two back translations conducted by a professional translation company. These were followed by discussions involving the translators, the original developers, and the Norwegian in-country expert team to ensure conceptual equivalence with the original. Cognitive debriefing interviews were conducted with LTC users. The introduction and instructions for interviewers and respondents were adapted to fit the Norwegian context.

**Results:**

The initial translations were overly literal. Based on extensive team discussions and findings from cognitive interviews, the text was revised to achieve fluent, simple, everyday Norwegian language in the instructions, questions and response options. Any superfluous text was removed.

**Conclusion:**

The Norwegian version of the ASCOT INT4 is a carefully translated and culturally adapted instrument that closely reflects the constructs of the original English version.

## Introduction

Quality improvement in health and care services has received significant attention in Norway [1], with recent policies placing increased emphasis on quality of life **(QoL)** as an outcome of care service delivery for older people [2, 3]. Although these policies have contributed to improvements in several areas [4], there remains a need to strengthen person-centred care and QoL in long-term care (LTC). Capturing outcomes of LTC is therefore a policy priority in Norway, as in other European countries [5]. In specialist health services, user surveys assessing experiences among different patient groups are well established, for example in general hospitals, maternity care, and mental health care [6–8]. Results from these surveys are fed back to providers to support quality assurance and bench marking, and they often include assessments of treatment experiences as well as outcomes such as self-rated health and QoL.

In Norwegian municipal health and care services, however, there is no standardized approach for capturing the experiences or outcomes of people using LTC. Surveys are conducted but vary in content and frequency [9] and lack appropriate outcome measures. LTC users represent a diverse group whose needs arise from a wide range of health conditions and life situations. Nonetheless, maintaining the best possible QoL under their individual circumstances is a shared goal. The World Health Organization defines QoL as an individual's perception of their position in life in the context of the culture and value systems in which they live and in relation to their goals, expectations, standards and concerns [10]. An outcome measure that links the users' perception of their achieved QoL to the LTC services they receive would therefore be a valuable contribution for evaluation purposes**.**

The Adult Social Care Outcomes Toolkit (ASCOT) was developed in England for this purpose and captures those aspects of QoL most affected by LTC services [11]. ASCOT is used to measure Social Care-Related Quality of Life (SCRQoL) across a wide range of LTC settings, including home care and care homes [12]. It can be applied in needs assessments and care planning [13, 14] and as a preference-based measure in economic evaluations [11]. Since its launching 2012, it has been translated into several languages including German [15], Finnish [16], Dutch [17], Danish [18], Japanese [19], and Hong Kong Chinese [20], with accredited translations also available in Spanish and Swedish [21]. The toolkit composes eight distinct domains: control over daily life, personal cleanliness and comfort, food and drink, safety, social participation and involvement, occupation, accommodation cleanliness and comfort, and dignity [11]. Each domain has four response options, representing states ranging from an ideal state, no unmet needs, some unmet needs, and high level of unmet needs, in which the person’s physical or psychological health is at risk. Raw scores can be preference-weighted using population-specific preference weights developed in the UK [11], Austria [22], Finland [23] and Japan [24].

ASCOT includes a suite of tools targeting a range of adult groups that receive LTC, including interview-administered versions, self-completion questionnaires, proxy-questionnaires, Easy-Read versions, and a mixed-methods version for use in care homes [12]. All versions capture *current SCRQoL* (the person’s SCQoL at the time of reporting) in each domain. In the interview tool (INT4) [11] and the mixed-methods tool for use in care homes (CH4) [25, 26] the current score is followed by a second question asking whether the services they receive have an impact on the domain, and if the answer is affirmative, a third question about what they expect the state would be in the absence of services in the domain, the *expected SCRQoL* [27]. The “SCRQoL Gain” (current score–expected score) represent the impact of services on the individual’s SCRQoL [11]. ASCOT emphasizes individual preferences and includes both basic (e.g. food and drink, safety) and higher order domains (e.g. control and social participation) [28]. In doing so, it goes beyond a focus on health status, which is the core of most established QoL instruments [11, 29, 30].

Given the lack of standardized measures that capture QoL outcomes of LTC in Norway, the objective of this study was to translate and culturally adapt the face-to-face structured interview tool ASCOT-INT4 from English into Norwegian, achieving conceptual equivalence with the original.

The qualitative rationale for pursuing conceptual equivalence in translation rests on preserving the authenticity and integrity of the underlying constructs across cultures, leaving the translation process an interpretive process capturing culturally embedded meanings to maintain trustworthiness and validity across settings. In such, the richness of possible meanings within any utterance means translation is never one-to-one; instead, it demands reflexive critique and dialogue to uncover whether the translated text faithfully represents the original intent or subtly reshapes it [31]. Without conceptual equivalence, differences in response may reflect cultural interpretations rather than variations in the phenomenon of interest.

In this paper, we document the qualitative rationale and methodology used to translate and adapt the ASCOT tool for use in a range of LTC settings in Norway.

## Methods

### Setting

In Norway, the delivery of health and care services is divided between municipal health and care services and specialist health services, the latter operated by state-owned health enterprises. Municipalities are responsible for LTC, delivered to people who are assessed as being dependent on help due to physical or mental impairment. Services are defined by the Health and Care Services Act and include home health care and practical assistance. They may be in the home in nursing homes or in other institutional settings such as short term stays, respite care or sheltered housing [32]. Home health care may include services such as home nursing, mental health care, physiotherapy, or rehabilitation. Practical assistance may involve assistance in daily tasks, training for coping with daily tasks or user-controlled personal assistants. Practical assistance corresponds closely with social care in England. “*Examples of practical assistance include: help with shopping; cooking; washing of clothes and house cleaning; snow clearance; help with self-care”* [33]. A service allocator in the municipality decide whether needs falls under home health care or practical assistance [33]. Municipalities are free to organize and deliver care services according to local needs, resources, and priorities [34, 35]. In 2024, a total of 181 992 citizens aged 67 and older used municipal health and care services nationwide. Among them, 15 543 received only practical assistance, 55 471 received only home health care, and 41 553 received both. Additionally, 35 222 individuals stayed either long-term or short-term in institutions, while 45 677 received "other services" while living at home [36].

### Translation process

The translation process began in March 2021 and was completed in June 2022. The ASCOT instrument was first reviewed for relevance in the Norwegian context. Four groups participated in the process: the original instrument developers (ASCOT team from England), a professional external translation company (RWS, https://www.rws.com/), the Norwegian in-country expert team and the informants who participated in cognitive debriefing. The translation followed guidelines recommended by the developers [37] and was based on a ten steps procedure outlined by Wild et al. [38], previously used in translations of ASCOT into German, Japanese and Hong Kong Chinese [19, 20, 39]. The translation work was carried out collaboratively by bilingual team members to ensure interpretive integrity and to maintain conceptual equivalence across cultures as wording was adapted. This approach safeguarded content relevance and helped prevent culturally skewed interpretations. The procedure aligns with established practice for conducting translation, adaptation, and cross-cultural validation [40].

To support conceptual equivalence, the developers’ concept elaboration guide was used throughout the process. The guide provided detailed explanations of the constructs underlying each ASCOT domain and was consulted repeatedly during translation and adaptation.

Forward and back translation with reconciliations was carried out by the translation company. These initial translations were highly literal and served as the starting point for a more extensive adaptation process aimed at producing clear, everyday Norwegian language tailored to the respondent groups, while preserving the original conceptual meaning. This work was again carried out collaboratively by bilingual team members to ensure accuracy and cultural appropriateness.

Steps in the translation process were documented in a translation table with elements in the instrument in rows and input from stages in the process in columns (Table 1).

**Table 1:** Excerpts from the translation table [ © University of Kent, 2024, all rights reserved]

Source text	Harmonized translation	Discussion points from translators, in-country team, or developers	Final Norwegian
Thinking about keeping clean and presentable in appearance, which of the following statements best describes your situation?	Når du tenker på å holde deg ren og presentabel av utseende, hvilken av følgende uttalelser beskriver best din situasjon?	"in appearance" should be left out, appearance is given by nature and not only a positive or negative result of keeping clean, old age and frailty may make people perceive themselves as less presentable Clause containing the central terms should end the sentence	Hvilken av de følgende setningene beskriver best din situasjon med hensyn til å føle deg ren og velstelt?
I feel clean and am able to present myself the way I like	Jeg føler meg ren og er i stand til å vise meg på den måten jeg liker		Jeg føler meg ren og velstelt slik jeg selv liker det
I feel adequately clean and presentable	Jeg føler meg tilstrekkelig ren og presentabel		Jeg føler meg ganske ren og velstelt
I feel less than adequately clean or presentable	Jeg føler meg mindre enn ren og presentabel	Please include “adequately” in the translation → Jeg føler meg mindre enn adekvat ren og presentabel	Jeg føler meg ikke tilstrekkelig ren og velstelt
I don’t feel at all clean or presentable	Jeg føler meg ikke ren og presentabel i det hele tatt		Jeg føler meg slett ikke ren og velstelt
6. Imagine that you didn’t have the support and services from [–-] that you do now and no other help stepped in. Which of the following would then best describe your situation with regard to keeping clean and presentable in appearance?	6. Tenk deg at du ikke hadde støtten og tjenestene fra [–-] som du har nå og ingen annen hjelp trådde til. Hvilke av følgende ville da best beskrive din situasjon med hensyn til å holde deg ren og presentabel av utseende?		Tenk deg at du ikke fikk den hjelpen som du får nå, og ingen andre hjalp deg. I en slik tenkt situasjon, hvilken av de følgende setningene ville da (ha) vært den beste beskrivelsen av din situasjon med hensyn til å føle deg ren og velstelt?
Interviewer note:	Merknad for intervjuer:		Merknad for intervjuer:
(…)	(…)	(…)	(…)
*Reassure if necessary:* Please be assured that this is purely imaginary and does not affect the services you receive in any way	*Berolige om nødvendig:* Vᴂr forsikret om at dette er rent imaginᴂrt og innvirker ikke på noen måte på tjenestene du får	Language improvement needed	*Berolige om nødvendig:* Dette er helt og holdent en tenkt situasjon og svarene du gir vil ikke på noen måte ha noe å si for tjenestene du mottar
(…)	(…)	(…)	(…)
I would feel less than adequately clean or presentable	Jeg ville føle meg mindre enn tilstrekkelig ren eller presentabel	Language improvement needed	Jeg ville ikke (ha) følt meg tilstrekkelig ren eller velstelt

**Fig. 1:**
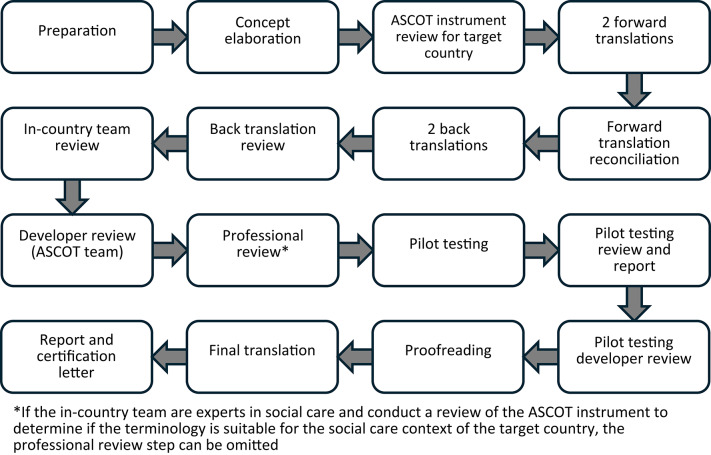
Stages of translation and cultural adaptation process of the ASCOT toolkit (ASCOT team 2025, adapted from Wild et al., 2005)

The in-country team was highly qualified to contribute to the process (Fig. 1). The majority of the nine members held a Ph.D.-degree with expertise in health services research. The members’ linguistic English—Norwegian skills ranged from proficient to bilingual. Four members were registered nurses with clinical LTC experience, making the team particularly suited to review terminology and ensure contextual fit.

Cognitive debriefing was conducted to identify an resolve potential issues related to the survey format, contents, or wording among intended respondents. Ideally, these interviews apply a ‘think aloud’-technique and-or verbal probing. [41]. However, at the planned time for these interviews (January 2022), strict COVID-19 restrictions limited contact with frail older adults, which was our primary target group for recruitment. As a second-best approach, cognitive interview initially included users living at home who were able to complete interviews via video call. Once the contact restrictions were lifted, we could add face-to-face interviews (May 2022).

### Ethical considerations

Healthcare workers recruited informants for cognitive debriefing. To minimize formalities associated with privacy protection, we collected only minimal background data about these respondents. They received written information about interview purposes, their rights, and that recordings would be transcribed and then deleted. The Data Protection Officer at the Norwegian Institute of Public Health found the personal data limited and not requiring ethical approval. The large project of which the translation was a part was approved by the Regional Committee for Medical and Health Research Ethics (ref nr 257,661).

## Results

The analysis to evaluate the relevance of ASCOT in the Norwegian context showed it to align well with the statutory and regulatory framework governing municipal health and care services in Norway. Both the *Regulation on Quality in Care Services (kvalitetsforskriften)* and the *Health and Care Services Act (lov om kommunale helse- og omsorgstjenester)* emphasize person-centred, safe, and individually adapted services that enable users to maintain autonomy, participate in meaningful activities, and live a dignified life. Importantly, lower-order domains such as access to adequate food and support with personal hygiene are explicitly identified as fundamental responsibilities of municipal services and constitute prerequisites for maintaining health, comfort, and personal integrity. By capturing these basic needs alongside higher-order aspects of participation and autonomy, the measure provides a comprehensive framework for assessing whether municipal services fulfil legal expectations and promote holistic wellbeing among service users.

Following the initial translation, the in-country team of experts judged that the word-for-word translation provided by the translation company needed substantial editing to reflect both the original concept and Norwegian everyday spoken language in the intended population. Consequently, the in-country team of experts critically discussed the wording and content of the original text, and how to revise the Norwegian text to maintain conceptual equivalence with the original ASCOT instrument. The harmonization included repeated lookups in the concept elaboration guide, considering translations of the items in other known languages (Danish, Dutch and German) as well as discussions with the ASCOT team. This led to the following initial adaptations.

The opening interviewer notes and instructions were adapted to fit the Norwegian context where terms had no Norwegian counterparts, for example “personal budget”. To adjust the instruction to the Norwegian care context, where health and social care are delivered in an integrated manner in accordance with the obligation of the municipal health care services, the instruction for the respondent not to include *“support from health professionals, such as doctors/GPs, nurses or physiotherapists”* was omitted. Instead, the Norwegian respondents were instructed *to “think of the services you receive from the municipality, it could be home nursing care, practical assistance, adult day care, sheltered housing, or a place in a nursing home”.*

Some key terms used repeatedly in the original version were challenging when translated literally and thus were discussed with the instrument developers. For example, in six of the domains “Adequate/adequately” is used repeatedly to express the “no needs”-state among the response options. However, in Norwegian, the adjective "adequate" is not an everyday word, and the adverb "adequately" does not exist. Therefore, it was translated to “tilstrekkelig” (sufficiently) or “nok” (enough). Also important was the discussion about how to best capture in everyday Norwegian the verb “affect” in the second question posed for each domain, “Do the support and services you get from [services] affect ….” The professional translation agency suggested ‘innvirke på’. However, ‘innvirke på’ has a rather mechanical connotation and was replaced with’Do the support and services you get from [service provider] mean (= “betyr”) anything for… [domain]…’. Of less importance was, for example the word ‘prompt’ that is used repeatedly in instructions to the interviewer. There is no Norwegian counterpart, and we found ‘tilføyelse’ (≈ addition) to be a serviceable replacement.

### Counterfactual questions: expected SCRQoL

The third question in each domain asked the respondent what they would expect their status to be in the absence of care services, i.e., should they not have the support from the municipality, and no other help stepped in. Such hypothetical questions are considered cognitively demanding, as they require informants to imagine detailed non-existing scenarios that differ from their actual current situation [42, 43] and hence require precise wording. Modern Norwegian lacks a simple way to express counterfactual situations (subjunctive). Preteritum futurum perfectum is used in conditional sentences to emphasize the hypothetical or unreal in the condition. These forms vary between formal writing and everyday usage, and are used to express hypothetical situations, desires, hopes, or dreams, especially when discussing unlikely or speculative events. The auxiliary verb "ha" was thus made optional (parenthesized) in questions and answers pertaining to the counterfactual situation.

### Cognitive interviews

The video call informants were recruited through personal contacts, and the remaining were recruited by personnel at day care centres (Table 2).

**Table 2:** Informant characteristics

Sex	Age	Care need level	Mode	Dwelling
Female	70–80	Low	Video call	Own home
Male	50–60	Low	Video call	Own home
Female (spouse)	80–90	Moderate	Face to face	Sheltered housing
Male (spouse)	80–90	Low	Face to face	Sheltered housing
Female	80–90	High	Face to face	Sheltered housing
Female	80–90	High	Face to face	Sheltered housing
Female	80–90	Low	Face to face	Sheltered housing

The cognitive face-to-face interviews lasted for 32 to 68 min. Most informants were frail, and it was difficult to establish and maintain a strict "think aloud" procedure. The face-to-face interviews developed into conversations about the content of the domains and response options, in relation to the informants' daily lives. The conversations were directed with verbal probing, though carefully, as steering the conversation strictly towards the interviewers' specific goals and needs could be perceived as intrusive.

Despite the need for methodological flexibility (for example, no strict "think aloud" procedure), conversations and discussions with the informants showed that they recognized the domains from their own daily lives and that the response scales were perceived as ordered states of quality of life. The cognitive interviews also revealed that the three questions and eleven response options per domain were wordy. The most frail informants tended to lose track of the subject matter when listening to the rather similar response statements. Hence, it was an overall goal to keep the questions and response options as simple and clear as possible, and any superfluous words should be left out. When the sentences consisted of multiple clauses, we made sure that the meaning-bearing clause concluded the sentence, to help maintain focus.

### Linguistic adaptation

#### Domain: control over daily life

In this first domain the set of questions and response options seemed to work fine. As “control” may have different connotations, we recommend for the interviewer to use the suggested prompt from the original version of ASCOT by default: *“By “control over daily life” we mean having the choice to do things or have things done for you as you like it and when you want”.*

#### Domain: personal cleanliness and comfort

This is an example of moving the meaning bearing clause to the end. From *“Thinking about keeping clean and presentable in appearance, which of the following statements best describe your situation?”* to *“Which of the following statements best describe your situation regarding feeling clean and well-groomed?”* We replaced "presentable" with "well-groomed". Well-groomed is more in line with everyday language and effectively covers the English "presentable".

#### Domain: food and drink

The question was reworded from *“Thinking about the food and drink you get, which of the following statements best describes your situation?”* to *“Which of the following statements best describes your situation regarding food and drink?”.*

The response statements about food and drink allow for various interpretations, for example, level 2: “*I get adequate food and drink at OK times”.* This may pertain to quality, quantity, or timing and may appear unclear in both the Norwegian translation and the original. For some users living at home, the food may be delivered and kept, and the meal initiated and prepared by the users themselves at a convenient time. Such ambiguities may blur the differences between the status levels in some cases. However, the respondents recognized the underlying structure of the states in the response scale, and their answers seemed to be based on the order of the response options as much as on the exact wording.

#### Domain: personal safety

As “safety” may have different connotations, we recommended for the interviewer to use the suggested prompt by default: *“By “feeling safe” we mean how safe you feel both inside and outside the home. This includes fear of abuse, falling or other physical harm”.* To simplify, we changed response level 2 from “*Generally I feel adequately safe, but not as safe as I would like*” to “*I generally feel safe*”.

#### Domain: social participation

The question wording was simplified, from “*Thinking about how much contact you have with people you like, which of the following statements best describes your social situation?”* to *“Which of the following statements best describes your situation when you think about how much social contact you have?”* Except for in the first response option, “*I have as much social contact as I want with people I like*”, “*Social contact with people*” was simplified to “*social contact*”.

#### Domain: occupation/activity

We changed the heading from "occupation" to "activities" because the direct translation of "occupation" to Norwegian is associated with profession or working life. The Norwegian word for “activities” is inclusive of both paid work and leisure, which was considered by the expert team to be closer to the original concept in English. The introductory question deals with how the respondents spend their time and is unchanged from the original version. However, the response options introduce also to what extent they do the things they value and enjoy with their time, hence, it is unclear whether this is about the amount of time passing in passivity, or about the opportunity to do things they enjoy. The ambiguity of this domain is kept in the Norwegian translation, but for simplicity “*things I value and enjoy*” is replaced with “*things I like*”.

#### Domain: accommodation cleanliness and comfort

We added clarification that the question was about where they currently live. “Comfortable” was translated to “trivelig” as this is a more common term with which to describe well-functioning and pleasant daily accommodation, and “comfortable” evokes associations more in the direction of luxury. Our solution is similar to the German version, which uses “wohnlich” for comfortable.

#### Domain: dignity

In this final domain we added a clarification on the content of the statements: *“with “selvfølelse” we mean how you think and feel about yourself”.* We feared a potential ambiguity in the final question, “…the way I’m helped and treated….” as being “treated" can mean how other people relate to you, but also means receiving medical treatment. However, this was not observed as a problem by the interviewers in the pilot data collection, as the term’s context connects it to qualities of the personal interactions and attitudes.

### Omitting screening question 2: the impact of services

In all domains but Dignity the second question in the original version screens for differences between the current and the expected status, and reads: "Does the support and services you receive from [service provider] affect [the relevant domain]?" In Norway, prior to receiving services and hence becoming eligible for the survey, all the respondents have been assessed by a service allocator as having unmet needs. Consequently, the likelihood that the services do not affect any domain of SCRQoL is minimal and little or no important additional information is obtained by this screening. We decided to omit the screening second question in the Norwegian version, thereby reducing the total number of questions from 23 to 16 and achieving a substantial reduction in response burden (Table 3).

**Table 3:** Domains, with examples of questions and response categories in English and Norwegian (Control over daily life and Dignity) INT4-version of ASCOT toolkit [© University of Kent, 2024, all rights reserved] [11]

Domains	Examples of questions and response options, original and translated
**Control over daily life:** The service user can choose what to do and when to do it, having control over his/her daily life and activities	
	**English original**
	*Current status*
	Which of the following statements best describes how much control you have over your daily life?
	Interviewer prompt: By ‘control over daily life’ we mean having the choice to do things or have things done for you as you like and when you want
	If needed, please prompt: When answering the question, think about your situation at the moment
	I have as much control over my daily life as I want
	I have adequate control over my daily life
	I have some control over my daily life, but not enough
	I have no control over my daily life
	*Expected status, without social care*
	Imagine that you didn’t have the support and services from [services] that you do now and no other help stepped in. In that situation, which of the following would best describe the amount of control you would have over your daily life?
	Interviewer note: (…) Reassure if necessary: Please be assured that this is purely imaginary and does not affect the services you receive in any way
	I would have as much control over my daily life as I want
	I would have adequate control over my daily life
	I would have some control over my daily life, but not enough
	I would have no control over my daily life
	**Norwegian translation**
	*Current status*
	Hvilken av de følgende setningene beskriver best hvor mye kontroll du har over hverdagen din?
	Tilføyelse for intervjuer: Med "kontroll over hverdagen" mener vi å kunne gjøre ting selv eller få ting gjort for deg slik du ønsker og når du ønsker det
	Om nødvendig, tilføy: Tenk på hvordan hverdagen din er nå for tiden
	Jeg har så mye kontroll over hverdagen min som jeg selv ønsker
	Jeg har tilstrekkelig kontroll over hverdagen min
	Jeg har litt kontroll over hverdagen min, men ikke nok
	Jeg har ingen kontroll over hverdagen min
	*Expected status, without social care*
	Tenk deg at du ikke fikk den hjelpen som du får nå, og ingen andre hjalp deg. I en slik tenkt situasjon, hvilken av de følgende setningene ville da (ha) vært den beste beskrivelsen av hvor mye kontroll du ville (ha) hatt over hverdagen din?
	Merknad for intervjuer: (…) Berolige om nødvendig: Dette er helt og holdent en tenkt situasjon og svarene du gir vil ikke på noen måte ha noe å si for tjenestene du mottar
	Jeg ville (ha) hatt så mye kontroll over hverdagen min som jeg ønsker
	Jeg ville (ha) hatt tilstrekkelig kontroll over hverdagen min
	Jeg ville (ha) hatt litt kontroll over hverdagen min, men ikke nok
	Jeg ville ikke (ha) hatt noen kontroll over hverdagen min
**Personal cleanliness and comfort:** The service user feels that he/she is personally clean and comfortable and looks presentable or, at best, is dressed and groomed in a way that reflects his/her personal preferences	
**Food and drink:** The service user feels that he/she has a nutritious, varied and culturally appropriate diet with enough food and drink that he/she enjoys at regular and timely intervals	
**Safety:** The service user feels safe and secure. This means being free from fear of abuse, falling or other physical harm, and fear of being attacked or robbed	
**Social participation and involvement:** The service user is content with his or her social situation, where social situation is taken to mean the sustenance of meaningful relationships with friends and family, and feeling involved or part of a community should this be important to him/her	
**Occupation:** The service user is sufficiently occupied in a range of meaningful activities, whether formal employment, unpaid work, caring for others or leisure activities	
**Accommodation cleanliness and comfort:** The service user feels that his or her home environment, including all the rooms, is clean and comfortable	
**Dignity:** The negative and positive psychological impact of support and care on the service user’s personal sense of significance	
	**English original**
	Which of these statements best describes how the way you are helped and treated makes you think and feel about yourself?
	The way I’m helped and treated makes me think and feel better about myself
	The way I’m helped and treated does not affect the way I think or feel about myself
	The way I’m helped and treated sometimes undermines the way I think and feel about myself
	The way I’m helped and treated completely undermines the way I think and feel about myself
	**Norwegian translation**
	Hvilken av de følgende setningene er den beste beskrivelsen av hvordan måten hjelpen blir gitt på påvirker selvfølelsen din?
	Måten jeg blir hjulpet og behandlet på gjør selvfølelsen min bedre
	Måten jeg blir hjulpet og behandlet på har ingen betydning for selvfølelsen min
	Måten jeg blir hjulpet og behandlet på gjør selvfølelsen min dårligere
	Måten jeg blir hjulpet og behandlet på ødelegger selvfølelsen min helt

## Discussion

In this study we followed established procedures for translating the English ASCOT INT4-tool to Norwegian**.** The ASCOT tool is research based and validated. Based on the relevance analysis of the tool in the Norwegian context we argue, according to the argument-based approach to validity [44] the Norwegian translation to be a valid measure. This is also confirmed in psychometric analysis [45].

The introductory text was adapted to the Norwegian context, and the screening question assessing weather services affected each domain was omitted in all but one domain. This reduced the number of questions from 23 to 16, without loss of information about current and expected SCRQoL.

The translation process began with forward and back translations, as recommended in established guidelines [38]. However, revising these literal translations highlighted the challenge of remaining faithful to the English source text while achieving conceptual equivalence in colloquial Norwegian, where grammar, syntax, and everyday expressions differ substantially.

It was therefore essential to move beyond literal translations to effectively capture the necessary nuances in the questions and response categories [19, 20, 30]. The most effective strategies for achieving this proved to be the detailed assessments and deliberations within the in‑country expert team, the insights gained through cognitive interviews, and ongoing dialogue with the ASCOT developers. Similar to findings from the German translation, we concluded that greater emphasis on conceptual rather than semantic equivalence earlier in the process would have been beneficial, and that the back‑translation step contributed less to overall translation quality [39, 46].

One recurrent linguistic challenge concerned the term "adequately" which appears frequently in ASCOT to indicate the “no unmet needs” state. Norwegian has no natural everyday equivalent, and similar difficulties have been reported in the German and Dutch translations, where the solution was to use terms corresponding to “enough” or “sufficiently” [17, 39]. Another well‑known challenge was translating the concept of dignity, which lacks a direct Norwegian equivalent. As in the Dutch, German, Chinese, and Japanese versions, which used terms such as “being respected,” “self‑confidence,” or “self‑esteem”.

[17, 19, 20, 39]. In Norwegian, we added a default prompt specifying that the question was about “self-feeling”, meaning how you think and feel about yourself. Based on the thorough discussion in the expert group, supported by the think aloud interviews, we argue that the translation process has produced a questionnaire that mirrors the original questionnaire very well.

### Adaptation

The most significant adaptation in the survey's format was the omission of screening questions from seven domains. A large proportion of the service recipients are frail, and this adjustment was made to balance the response burden with the value of the information thus provided [47]. The tool could have been further shortened by replacing the full sentences in the response categories with shorter, generic labels (e.g., Very high QoL, High QoL, Low QoL, Too low QoL). As did Nakamura et al. [19], we decided against this, as the measurements would no longer be standardised across countries.

Given the integration of health and social care service delivery in Norwegian municipalities, we omitted the original text instructing respondents not to consider support from health professionals when responding. This was replaced by a text instructing respondents to think about health and care services from the municipality, with examples given: *“home nursing care, practical assistance, adult day care, sheltered housing, or a place in a nursing home”.* A large proportion of the Norwegian LTC users receive home nursing only and a small proportion receive practical assistance only [36, 48]. In 2022, about half of Norwegian adults ≥ 67 years reported having chronic illness or health problems [49], and health authorities have stated that the municipalities are becoming ever more important providers of health and care services [50]. A study of contemporary policy documents found that the contribution of home care nursing is largely invisible for society and policymakers [51]. Hence, the absence in ASCOT of a domain to measure current and expected quality of life related to care aimed at health maintenance and/or medical interventions may represent a shortcoming in terms of content validity for some user groups in the Norwegian setting. The significance of this absence should be explored in future studies.

A Comparison of the LCT Systems in Norway and England showed that the two systems exhibit more similarities than differences. The most notable differences included funding mechanisms, the degree of care integration and implementation of national outcome measures. However, both countries have implemented similar initiatives to address shared challenges within their services like person-centred care and ageing in place policies (Keemink et al., submitted).

Even though cultural and health system differences can make constructs hard to compare across countries, the translated ASCOT tool seems to be a useful tool for cross country comparison, as it aligns well with OECD standards for evidence‑based cross‑country comparisons because it operationalises clearly defined, universally interpretable domains—such as control over daily life, personal cleanliness, food and drink, safety, and dignity—in a manner consistent with the OECD’s emphasis on conceptual clarity, relevance, and harmonisation. According to the OECD Statistical Quality Framework, high‑quality comparative indicators must be conceptually consistent, relevant, and built on shared definitions and user‑centred needs [52]. The instrument’s domains represent widely recognised components of wellbeing and service quality, and their structured definitions support harmonised measurement across national contexts. Moreover, the framework stresses that cross‑country comparisons require coherence and comparability across datasets, alongside clear metadata and transparent methodological documentation. Because the instrument uses well‑specified, observable dimensions of care—many of which correspond to internationally acknowledged basic care responsibilities—the measures can be consistently interpreted across countries and mapped onto existing OECD frameworks for social care and wellbeing. Finally, the OECD’s Recommendation on Good Statistical Practice highlights the importance of transparency, credibility, and methodological independence as prerequisites for high‑quality international comparisons. A structured, domain‑based social care related quality‑of‑life instrument supports these expectations by enabling clear documentation of definitions, data collection procedures, and scoring rules, thereby ensuring that cross‑national analyses rest on robust and transparent evidence.

## Conclusion

We argue that adherence to the established translation and adaptation procedures has ensured conceptual equivalence in the explanations, prompts, questions, and response options between the English original and the Norwegian version of ASCOT INT4. The Norwegian version employs clear everyday language and supports the collection of valid measures that allow for meaningful comparison across countries. The Norwegian version is the property of the University of Kent and can be made available by making contact via https://research.kent.ac.uk/ascot/.
